# Effect of Using Swiss Chard Powder as Alternative Curing Agent in Sucuk Production on the Growth of *Listeria monocytogenes*

**DOI:** 10.3390/foods15152728

**Published:** 2026-08-03

**Authors:** Bilge Ulutaş Ataylı, Zeynep Feyza Yılmaz Oral, Yağmur Akyol, Güzin Kaban, Mükerrem Kaya

**Affiliations:** 1 Quality Coordination Office, Rectorate, Artvin Çoruh University, Artvin TR-08010, Türkiye; 2 Department of Food Engineering, Faculty of Agriculture, Atatürk University, Erzurum TR-25240, Türkiye; zeynep.yilmaz@atauni.edu.tr (Z.F.Y.O.); yagmur.akyoll@hotmail.com (Y.A.); gkaban@atauni.edu.tr (G.K.); mkaya@atauni.edu.tr (M.K.)

**Keywords:** fermented sausage, sucuk, Swiss chard powder, *L. monocytogenes*, curing agent, residual nitrite

## Abstract

The aim of this study was to determine the effect of using Swiss chard powder (SCP) and pre-converted SCP as alternative nitrate and nitrite sources in sucuk (a Turkish dry fermented sausage) production on the growth of *Listeria monocytogenes* and some quality characteristics of the product. Sucuk batters were prepared based on four different curing applications (synthetic nitrate; SCP; synthetic nitrite; pre-converted SCP). The batters were inoculated with *L. monocytogenes* at a level of 10^4^ cfu/g. Sucuk groups containing nitrate were subjected to slow ripening, while sucuk groups containing nitrite were subjected to rapid ripening. After 3 days of fermentation, in both rapid and slow ripening, the groups containing SCP and pre-converted SCP showed higher pH values than the groups containing synthetic nitrate and nitrite. At the end of ripening, the a_w_ value was found to be below 0.90 in all groups. SCP or pre-converted SCP did not have a significant effect on residual nitrite content. Lactic acid bacteria showed good growth in all four groups. Lower *Micrococcus*/*Staphylococcus* counts were observed in rapid ripening compared to slow ripening. As ripening time progressed, the number of *L. monocytogenes* decreased in all groups, with a mean reduction of 1.23 log cfu/g. At the end of ripening, no difference was observed between the groups in terms of *L. monocytogenes* count. As a result, SCP or pre-converted SCP showed similar results to synthetic curing agents in the inhibition of *L. monocytogenes* in sucuk. In addition, these natural products did not cause significant changes in the overall characteristics of the product.

## 1. Introduction

Curing is defined as the process of treating meat with salt and nitrate and/or nitrite to ensure food safety and provide characteristic color and flavor [1,2]. Product type is the most important factor in selecting a curing agent. The main curing agent is nitrite. Therefore, in products using nitrate, this needs to be converted to nitrite in order to exhibit the expected effects of this curing agent [3,4,5]. Curing agents are widely used in the production of meat products, with the exception of fresh processed meat products and some traditional products [6]. Nitrite and nitrate, in addition to their positive effects in cured products, are also involved in the formation of N-nitrosamines [7]. Therefore, processed meat products are a major source of concern for consumers [7,8,9,10]. Due to these concerns, the use of nitrate-rich vegetable juices and powders as alternative curing agents has long been a focus [8,10]. However, the conversion of nitrate in vegetable extracts to nitrite in the product is essential for effective curing [11,12]. For this purpose, bacteria with nitrate reductase activity such as *Staphylococcus xylosus* and *S. carnosus* are used, thereby rendering these products a source of nitrite [12]. Vegetable extracts subjected to this process are termed “pre-converted” [12], “cultured”, or “pre-fermented” [11] extracts.

The effects of vegetable juices and powders (as natural nitrate sources) or their pre-converted forms (as natural nitrite sources) such as celery, Swiss chard, beet root, parsley, radish, leek powder, spinach and red beet on the physical, chemical and sensory properties as well as general microbiological characteristics of various types of fermented sausages such as chorizo, sucuk, heat-treated sucuk, salami, and Italian-style dry sausage have been investigated [13,14,15,16,17,18,19,20,21,22,23]. However, research on the effects of using vegetable-based curing agents in these products on the behavior of foodborne pathogens is quite limited [7,10,24].

*Listeria monocytogenes* is one of the most important foodborne pathogens in fermented sausages. This pathogenic microorganism is psychrotrophic and can grow over a wide pH (4.2–9.5) range [25]. The minimum a_w_ value for this microorganism is 0.92 [26]. Nitrate and/or nitrite are significant hurdles for the control of *L. monocytogenes* in fermented sausages [7,25,26,27]. Since these additives are synthetic substances, interest in plant-based curing agents is increasing day by day. Therefore, research on the effects of natural curing agents on the behavior/inhibition of pathogenic microorganisms in fermented sausages is of great importance [10].

Sucuk, which belongs to the group of dry fermented sausages, is widely consumed in Türkiye. In sucuk production, the initial fermentation temperature varies between 12–26 °C depending on the ripening rate. The ripening (fermentation and drying) time can extend up to 20 days [28,29]. Nitrate and/or nitrite are used as curing agents in their production, depending on the ripening rate. Nitrate is typically used in slow-ripened processes, whereas nitrite is preferred in rapid-ripened sausages. Furthermore, in slow ripening, initial fermentation temperatures below 20 °C are generally applied. In contrast, the initial fermentation temperature for rapid ripening typically ranges from 22 to 24 °C [6,29]. According to the Turkish Food Codex Regulation on Food Additives [30], sodium and potassium salts of nitrate and nitrite are permitted in the production of sucuk. The maximum permitted amount for both nitrate and nitrite is 150 mg/kg, expressed as NaNO_2_ or NaNO_3_. However, this regulation does not yet include any provisions regarding the use of vegetable-based curing agents. The regulatory status of vegetable extracts used as curing agents remains unclear in many countries. It is noted that this situation may cause confusion or mislead consumers. A plant extract added to a food to perform a technological preservation function is legally classified as an additive and must comply with the relevant EU regulations [11]. Nitrite has been shown to influence the reduction of *L. monocytogenes* during the ripening of sucuk [31]. Furthermore, it is assumed that lactic acid bacteria, especially bacteriocin-producing lactic acid bacteria strains, are the most important factor for the control of *L. monocytogenes* in this product in the presence of nitrite [32,33,34]. In addition, it was reported that the initial fermentation temperature plays an important role in the growth of *L. monocytogenes* [35]. Studies have also been conducted on the possibilities of using plant-based alternative curing agents in sucuk production [17,22,36]. However, to date, there is no study about the effect of plant-based alternative curing agents on the behavior of *L. monocytogenes* during ripening of sucuk. The aim of this study was to determine the effect of using Swiss chard powder (SCP) and pre-converted SCP as alternative nitrate and nitrite sources in sucuk (a Turkish dry fermented sausage) production on the behavior of *L. monocytogenes*. In addition, the influence of these natural curing agents on the pH and a_w_ values of sucuk, as well as on the numbers of lactic acid bacteria, *Micrococcus*/*Staphylococcus*, yeast-mold, and Enterobacteriaceae, were determined.

## 2. Materials and Methods

### 2.1. Material

In this study, large beef cuts (round) obtained from three different beef carcasses (48 h postmortem, pH < 5.9) were used as the raw material. Beef fat was also obtained from the same carcasses. The meat pieces from each carcass were divided into 4 batches after the coarse connective tissues were removed. The same process was applied to the meat fat. After the meat and fat were divided into batches, they were separately packaged, frozen, and stored at −18 °C until production. Autochthonous *Staphylococcus xylosus* GM92 [37] and *Latilactobacillus sakei* S15 [38] strains were used as starter cultures. Sucuk batter was artificially contaminated with two strains of *Listeria monocytogenes* (*L. monocytogenes* ATCC-19112 and *L. monocytogenes* ATCC-19114). *L. monocytogenes* strains were cultivated in TSB (Tryptic Soy Broth) (Merck, Darmstadt, Germany) medium at 37 °C for 24 h. *L. sakei* S15 MRS (de Man Rogosa Sharpe) (Merck) broth was cultivated at 30 °C for 24 h, while *S. xylosus* GM92 was cultivated in TSB at 30 °C for 24 h. A commercial Swiss Chard powder (SCP) (Veg Dry–166, nitrate content: 37,200 mg/kg, Florida Food Products, FFP Product Code-166, Lake Mary, FL, USA) was used as a natural nitrate source, and pre-converted SCP (Veg Stable–531, Swiss Chard, nitrite content: 14,570 mg/kg, Florida Food Products, FFP Product Code-531, Lake Mary, FL, USA) was used as a natural nitrite source. Sodium nitrite (NaNO_2_) and potassium nitrate (KNO_3_) were used as synthetic nitrite and nitrate, respectively.

### 2.2. Sucuk Production

For the preparation of the sucuk batter, 20 g of salt, 9 g of cumin, 10 g of garlic, 5 g of black pepper, 7 g of red pepper, 4 g of sucrose, and 2.5 g of allspice were used for 1 kg of lean beef and beef fat (8:2) [29]. In the study, four different treatments were applied (A: 150 mg/kg KNO_3_, B: 150 mg/kg KNO_3_ equivalent SCP (0.403 SCP g/kg), C: 150 mg/kg NaNO_2_, D: 150 mg/kg NaNO_2_ equivalent pre-converted SCP (1029 pre-converted SCP g/kg)). A cutter (Mado, Dornhan, Germany) was used to prepare the sucuk batters. For each treatment, 4 kg of meat and fat mixture (3.20 kg + 0.80 kg) was used. Each treatment group was inoculated with autochthonous *L. sakei* S15 (10^7^ cfu/g) and *S. xylosus* GM92 (10^6^ cfu/g) strains as starter cultures. The batter was then inoculated with two strains of *L. monocytogenes* strain cocktails at an initial number of 10^4^ cfu/g. After inoculation, the batter was filled into collagen casings (38 mm diameter, Naturin Darm, Weinheim, Germany) using a laboratory-type piston filler (Mado, Dornhan, Germany) (each sucuk 200 g). A climate control unit (Reich, Klima-Rauchertechnik, Stuttgart, Germany) was used for the ripening of the sucuk. The initial fermentation temperature was 18 ± 1 °C for groups A and B (slow ripening), and 24 ± 1 °C for groups C and D (rapid ripening). After one day, the ripening temperature in each pair of groups was gradually reduced to 14 ± 1 °C. At both fermentation temperatures, the relative humidity was maintained at 92 ± 2% on the first day. It was then gradually reduced to 84 ± 2% on subsequent days. Air flow was also gradually decreased, from rapid to slow. Three independent sucuk manufactures were carried out on different days (Table S1).

### 2.3. Physicochemical Analyses

#### 2.3.1. pH

A total of 10 g of sample was homogenized with distilled water (100 mL) in an ultra-turrax (IKA, Staufen, Germany). The pH value was determined using a pH meter (Mettler-Toledo, Greifensee, Switzerland).

#### 2.3.2. Water Activity (a_w_)

Water activity values were determined using an a_w_-meter (TH-500/a_w_ Sprint, Novasina, Lachen, Switzerland). The device was calibrated with salt solutions before use, and measurements were performed at 25 °C.

#### 2.3.3. Residual Nitrite

The method given by NMKL [39] was applied to determine the amount of residual nitrite. The residual nitrite content of the extracted samples was determined by HPLC/DAD (Agilent, Santa Clara, CA, USA). A Hamilton PRP-X100 column (5 µm × 150 × 4.6 mm, Reno, NV, USA) was used, and the UV wavelength was 220 nm. The injection volume was 100 µL, and the flow rate was 2 mL/min. Lithium borate buffer solution was used as the mobile phase. For validation, standard solutions with different nitrite concentrations of 5–30 mg/L were measured in five replicates. The mean nitrite recovery rates ranged from 99% to 102.68%, and the relative standard deviations ranged from 1.30% to 2.75%. The coefficient of regression (R^2^) for the standard curve was 0.9999. LOD (1.12 mg/kg) and LOQ (3.21 mg/kg) values were calculated using the calibration curve of nitrite.

### 2.4. Microbiological Analyses

For these analyses, 25 g of sample was weighed, homogenized in a stomacher with 225 mL of sterile physiological saline (0.85% NaCl (Merck)), and then dilutions were prepared using sterile physiological saline. The counts given in Table 1 were performed.

### 2.5. Statistical Analyses

In the study, type of curing agent (synthetic nitrate, natural nitrate, synthetic nitrite, natural nitrite) and ripening time (0, 1, 3, 5, 7, 9 and 11 days) were used as factors, and the study was conducted according to the randomised complete block design in a 4 × 7 factorial plan. All experiments were performed using three independent biological replicates. Each biological replicate was analyzed in duplicate (two technical replicates). The mathematical model used was:

Y_ijk_ = μ + b_i_ + t_j_ + r_k_ + (tr)_jk_ + e_ijk_

where Y_ijk_ is the response variable; μ is the overall mean; b_i_ is the block effect; t_j_ is the fixed effect of type of curing agent; r_k_ is the fixed effect of ripening time; (tr)_jk_ is the interaction effect of type of curing agent and ripening time; and e_ijk_ is random error.

Type of curing agent and ripening time factors, as well as their interactions, were evaluated as fixed effects, while replications were evaluated as a random effect. In each experiment, each parameter was measured in duplicate, and the mean value was calculated. The results were checked for normality and homogeneity of variances and subjected to a two-way ANOVA. Mean values of variables among groups were compared using Duncan’s multiple comparison test, and the statistical significance was set at *p* < 0.05. Results were expressed as mean ± standard deviation (SPSS version 24, Chicago, IL, USA).

## 3. Results and Discussion

### 3.1. pH, a_w,_ and Residual Nitrite

**Figure 1:**
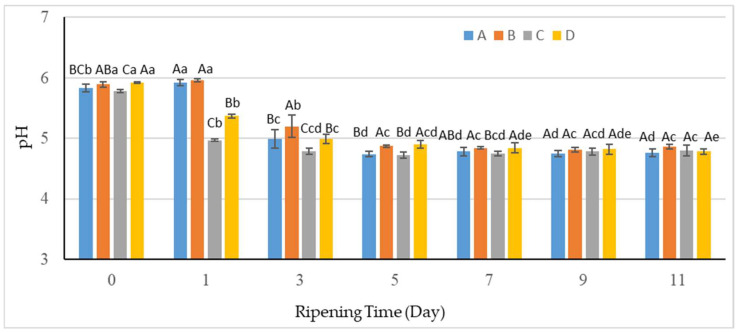
The effects of the interaction between type of curing agent and ripening time on the pH value. A–C: Different uppercase letters indicate significant differences between type of curing agent for ripening time. a–e: Different lowercase letters indicate significant differences between ripening time for type of curing agent. A: 150 mg/kg KNO_3_; B: 150 mg/kg KNO_3_ equivalent SCP; C: 150 mg/kg NaNO_2_; D: 150 mg/kg NaNO_2_ equivalent pre-converted SCP.

In fermented sausages, lactic acid bacteria, whether derived from the raw materials of the sausage mixture or added as starter cultures, cause a decrease in pH. They use carbohydrates to produce acids, primarily lactic acid. This decrease can vary depending on the rate and extent of acid formation by the lactic acid bacteria [6,42,43]. The fermentation rate in fermented sausages is determined by the curing agent used. When nitrate is used, slow fermentation is generally preferred because the gradual acidification enables microorganisms with nitrate reductase activity, particularly coagulase-negative staphylococci, to reduce nitrate to nitrite. Therefore, formulations containing synthetic or natural nitrate were subjected to slow fermentation, whereas sucuk batters containing synthetic or natural nitrite were processed under rapid fermentation conditions [6,11,29]. In this study, the type of curing agent had a very significant influence on the pH value. The lowest mean value was determined in the group with synthetic nitrite. The sucuk groups (C and D) cured with both synthetic and natural nitrite showed lower average pH values than the other groups (A and B), which can be attributed to the rapid ripening process applied to these groups (Table 1). Akköse et al. [29] also reported a faster pH drop in the first days of ripening during slow ripening (18 °C) compared to rapid ripening (24 °C). In the present study, ripening time also had a very significant effect (*p* < 0.01) on the pH value (Table 1). During the first three days of ripening, the mean pH value in all groups fell below 5.00. As Figure 1 shows, the lowest mean pH value in the sucuk batter (day 0) was measured in the group containing synthetic nitrite (group C). After one day of fermentation, groups A and B had higher mean pH values than groups C and D. Natural nitrite (pre-converted SCP) (group D) showed a higher pH value than synthetic nitrite (group C). This result is thought to be due to SCP increasing the pH of the sucuk batters. After 3 days of ripening, the lowest pH value was observed in the group with synthetic nitrite (group C). At the end of ripening, there was no statistically significant difference between the groups. Similarly, Hunt et al. [10] reported that the use of SCP in salami had no significant effect on pH.

**Figure 2:**
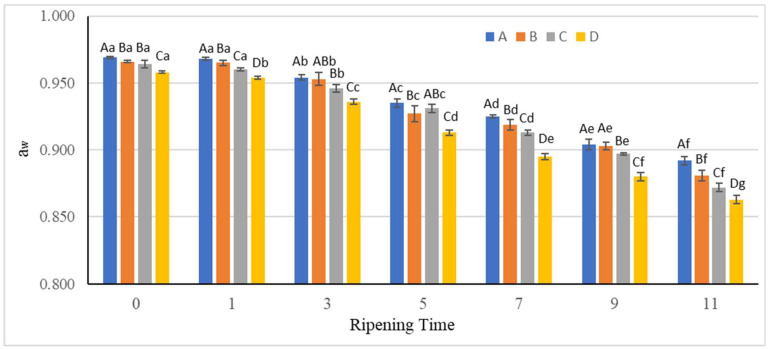
The effects of the interaction between type of curing agent and ripening time on the a_w_ value. A–D: Different uppercase letters indicate significant differences between type of curing agent for ripening time. a–g: Different lowercase letters indicate significant differences between ripening time for type of curing agent. A: 150 mg/kg KNO_3_; B: 150 mg/kg KNO_3_ equivalent SCP; C: 150 mg/kg NaNO_2_; D: 150 mg/kg NaNO_2_ equivalent pre-converted SCP.

In fermented sausages, the a_w_ value is considered a significant hurdle effect. The a_w_ value of fermented sausages such as sucuk is below 0.90 [6,44]. The a_w_ value was affected by the type of curing agent. Groups A and B, cured with synthetic nitrate and SCP, respectively, and undergoing slow ripening, showed higher mean a_w_ values than the rapidly ripened groups (C and D). The SCP group exhibited a higher mean a_w_ value than the synthetic nitrate group, and the pre-converted SCP group showed a higher mean a_w_ value than the synthetic nitrite group (Table 1). It is assumed that the lower a_w_ value in sucuk batters cured with SCP or pre-converted SCP is due to the water-binding properties of these powdered preparations. In fact, Yılmaz Oral [23] also found that the use of SCP in heat-treated sucuk resulted in a lower a_w_ value. Conversely, the a_w_ value decreased with increasing ripening time (Table 1). This is due to the decrease in moisture content. Similar results have been reported in previous studies [28,45]. As shown in Figure 2, the initial a_w_ value is above 0.95 in all groups. A similar decrease is observed in groups A, B, and C as the ripening period progresses. In contrast, a statistically significant decrease in the a_w_ value was found in the group containing pre-converted SCP after one day of fermentation. The lowest value at the end of ripening was also determined in this group. However, the a_w_ value was found to be below 0.90 in all treatments. According to EC Regulation [46], the growth of *L. monocytogenes* is not supported in ready-to-eat foods with a pH ≤ 4.4, or an a_w_ ≤ 0.92, or with a pH ≤ 5.0 in combination with an a_w_ ≤ 0.94. Accordingly, the final a_w_ value (<0.90) achieved in the products of the present study is sufficient to inhibit the growth of *L. monocytogenes* throughout shelf life, regardless of the use of synthetic or natural nitrate and/or nitrite.

The residual nitrite in meat products can vary depending on numerous factors such as pH value, storage conditions, processing temperatures, the presence of reducing agents, and the water content of the meat [47]. Residual nitrite levels were found to be below 10 mg/kg in all groups (Table 2). Residual nitrite levels are considered an important factor in nitrosamine formation [48]. As can be seen from Table 2, the type of curing agent had no significant effect on the residual nitrite content. These findings suggest that the source of the curing agent does not significantly influence residual nitrite levels and, consequently, nitrosamine formation. In all treatment groups, an autochthonous lactic acid bacteria strain was used as a lactic starter culture, and significant reductions in pH were recorded. The pH decrease during ripening accelerates the conversion of nitrite to nitric oxide. Thus, the amount of residual nitrite is significantly reduced [3,49,50]. Similar results have been reported in studies on sucuk produced with celery powder and heat-treated sucuk produced with chard powder [23,36].

### 3.2. Lactic Acid Bacteria, Microccoccus/Staphylococcus, and Enterobacteriaceae

**Figure 3:**
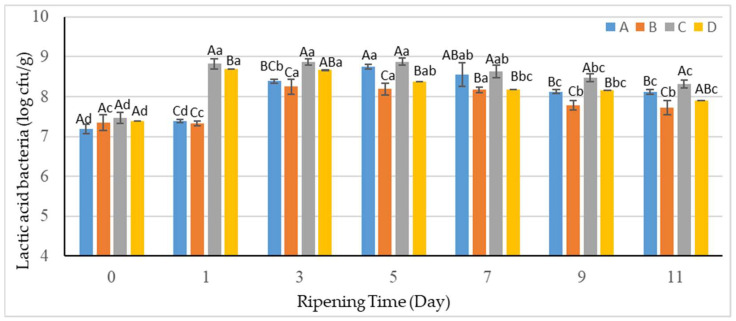
The effects of the interaction of using type of curing agent and ripening time on the lactic acid bacteria counts. A–C: Different uppercase letters indicate significant differences between type of curing agent for ripening time. a–d: Different lowercase letters indicate significant differences between ripening time for type of curing agent. A: 150 mg/kg KNO_3_; B: 150 mg/kg KNO_3_ equivalent SCP; C: 150 mg/kg NaNO_2_; D: 150 mg/kg NaNO_2_ equivalent pre-converted SCP.

Lactic acid bacteria and Gram-positive, catalase-positive cocci (*Micrococcus*/*Staphylococcus*) are technologically important microorganisms in fermented sausages [42]. In this study, the type of curing agent had a highly significant effect (*p* < 0.01) on the number of lactic acid bacteria. In groups C and D, the number of lactic acid bacteria was higher compared to the other groups due to the higher initial fermentation temperature. The ripening period also had an effect on these microorganisms (Table 3). As can be seen in Figure 3, no significant difference was determined between the groups in the sucuk batters. This result indicates that the *L. sakei* S15 strain was added to the sucuk batters at the same level. After one day of fermentation, groups C and D showed higher numbers than groups A and B. While there was no significant difference between synthetic nitrite and pre-converted SCP, synthetic nitrite exhibited slightly higher values than natural nitrite. On the third day of ripening, the difference between groups cured with nitrate (synthetic or natural) was not statistically significant. On the same day, there was also no significant difference between nitrite groups. However, in all groups, the lactic acid bacteria count is above 1 × 10^8^ cfu/g (Figure 3). In fermented sausages, fermentation is a critical stage. During this stage, the growth of lactic acid bacteria and consequently acid production are of great importance for the inhibition of pathogenic microorganisms [26]. At the end of the ripening process, the group cured with SCP-derived nitrate exhibited a lower mean value than the group treated with synthetic nitrate. Similarly, when nitrite was used, the pre-converted SCP group (natural nitrite) also showed a lower mean value than the synthetic nitrite group. However, in all groups, the lactic acid bacteria showed good growth during fermentation and reached high numbers. Similarly, a study on salami reported that in groups using chard and carrot juice concentrate powder as natural nitrate sources, lactic acid bacteria showed good growth during fermentation, and, at the end of ripening, similar results were obtained to those of the control group (synthetic nitrate/nitrite) [7]. These lactic acid bacteria counts are considered technologically sufficient [42].

**Figure 4:**
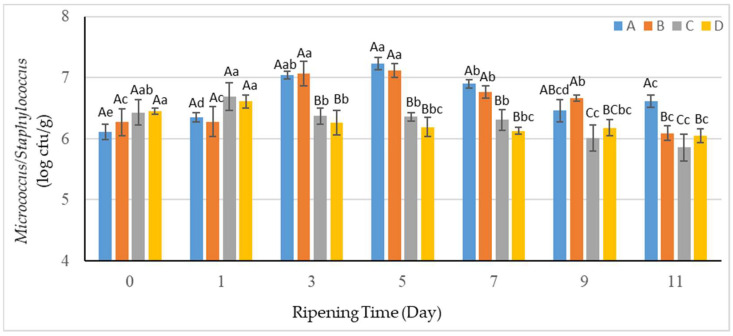
The effects of the interaction between type of curing agent and ripening time on the *Micrococcus*/*Staphylococcus* counts. A–C: Different uppercase letters indicate significant differences between type of curing agent for ripening time. a–e: Different lowercase letters indicate significant differences between ripening time for type of curing agent. A: 150 mg/kg KNO_3_; B: 150 mg/kg KNO_3_ equivalent SCP; C: 150 mg/kg NaNO_2_; D: 150 mg/kg NaNO_2_ equivalent pre-converted SCP.

The type of curing agent had a significant influence on the count of *Micrococcus*/*Staphylococcus*. The nitrate-cured groups (A and B) showed higher mean *Micrococcus*/*Staphylococcus* counts than the nitrite-cured groups (C and D) (Table 3). This result is attributed to the lower initial fermentation temperature (18 °C) in groups A and B and the resulting slower acidification [29,42]. The highest mean *Micrococcus*/*Staphylococcus* count was determined after five days of ripening (Table 3). The interaction between the type of curing agent and the ripening time had a very significant effect on the *Micrococcus*/*Staphylococcus* count in the sucuk (Table 3). As can be seen in Figure 4, no significant difference was observed between sucuk batters. This result is due to the addition of *S. xylosus* GM92 to sucuk batters at a level of 10^6^ cfu/g. A similar situation was determined after one day of fermentation. After 3 days of ripening, groups A and B showed higher numbers than groups C and D. This result is thought to stem from the use of nitrate in groups A and B. Indeed, Hospital et al. [51] also reported that nitrate promoted growth in these microorganisms. In addition, an excess of nitrite has been found to inhibit these microorganisms. It is also known that a slower decrease in pH during fermentation promotes the growth of these microorganisms [29]. In this study, acidification was slower in the nitrate-containing groups because the initial fermentation temperature was kept at 18 °C. The use of SCP or pre-converted SCP had no effect on the number of these microorganisms. Similar results were observed on days 5 and 7 of ripening. In contrast, at the end of ripening, the lowest *Micrococcus-Staphylococcus* count was determined in the treatment that used synthetic nitrite. However, the number did not fall below the inoculation level in any group. This result indicated that the *Staphylococcus xylosus* GM92 strain used showed good adaptation in all treatment groups, regardless of the type of curing agent.

*Enterobacteriaceae* are generally considered an indicator of possible microbiological contamination during processing [52,53]. In all sucuk batters (except for two samples), the number of *Enterobacteriaceae* was found to be below the detectable limit (<2 log cfu/g). After one day of fermentation and on subsequent days, the number of Enterobacteriaceae in all groups was below the detectable level. Nitrite acts as a significant hurdle against *Enterobacteriaceae* in fermented sausages [1,54]. These findings suggest that, in the nitrate-treated groups (A and B), *S. xylosus* GM92, used as the starter culture, converted nitrate to nitrite via its nitrate reductase activity, resulting in sufficient nitrite levels. Conversely, it has been reported in other studies that members of the *Enterobacteriaceae* family are generally found in sausages in numbers less than 100 cfu/g [29]. *Enterobacteriaceae* are also generally microorganisms that are sensitive to acidity [55]. Acidification caused by lactic acid bacteria is an important hurdle effect in inhibiting the growth of *Enterobacteriaceae* [53,56]. In addition, a_w_ is also an important hurdle effect factor for these microorganisms [34].

### 3.3. Listeria monocytogenes

**Figure 5:**
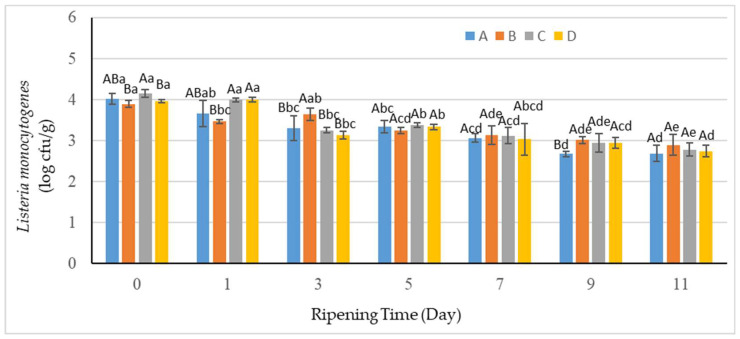
The effects of the interaction between type of curing agent and ripening time on the *Listeria monocytogenes* counts. A–B: Different uppercase letters indicate significant differences between type of curing agent for ripening time. a–e: Different lowercase letters indicate significant differences between ripening time for type of curing agent. A: 150 mg/kg KNO_3_; B: 150 mg/kg KNO_3_ equivalent SCP; C: 150 mg/kg NaNO_2_; D: 150 mg/kg NaNO_2_ equivalent pre-converted SCP.

*Listeria monocytogenes* is an important pathogen for sucuk, as well as for other fermented sausages [25,26,33,35]. In this study, the type of curing agent had no significant effect on the number of *L. monocytogenes* (Table 4). Nitrate alone is not a barrier to inhibiting foodborne pathogens in fermented sausages. For nitrate to be effective, it must be converted to nitrite. In this study, the *S. xylosus* GM92 strain was used as a starter culture in groups cured with nitrate (synthetic or natural). Due to the initial fermentation temperature applied in these groups, the pH did not drop rapidly initially, and this strain converted nitrate to nitrite through nitrate reductase activity [6]. Golden et al. [57] also reported that synthetic nitrite and nitrite derived from plant powder had a similar effect on *L. monocytogenes* in turkey breast (for delicatessen products) during refrigerated storage. Another study reported that dried cherries in cured-cooked sausage, after 10 days of cold storage, exhibited similar results to the control group in terms of *L. monocytogenes* behavior [58]. Riel et al. [59] reported that the inhibition rate of *L. monocytogenes* increased as the proportion of parsley extract powder increased in mortadella-type sausages (a type of emulsified sausage). In a study on salami, a similar reduction (>2 log cfu/g) in the number of *L. monocytogenes* was reported between groups containing synthetic nitrite and chard powder [10]. Ripening time significantly affected the number of *L. monocytogenes* (*p* < 0.01). A decrease in the mean number was observed during the ripening period. However, no statistically significant difference was found between the sucuk groups ripened for 3 and 5 days. A similar situation was observed on the 9th and 11th days of ripening. The mean number in the final product was determined as 2.77 log cfu/g. Accordingly, there was a mean reduction of 1.23 log cfu/g in the number of *L. monocytogenes* at the end of ripening. However, changes during ripening were affected by the type of curing agent (Table 3). As can be seen in Figure 5, synthetic nitrite and pre-converted SCP showed similar results after one day of ripening. Similarly, no difference was observed between the synthetic nitrate and SCP groups at the same ripening time. No differences were detected between all treatment groups on the 5th and 7th days of ripening. Similar results were observed at the end of ripening as well. These results show that there are no significant differences between synthetic curing agents and chard powder-derived curing agents in terms of *L. monocytogenes* inhibition. A study using Swiss chard juice concentrate powder and carrot juice concentrate powder (2.2 g/kg) as natural nitrate sources in salami found a significant reduction in *L. monocytogenes* numbers. However, the same study also reported that synthetic nitrate/nitrite (150/125 mg/kg) caused a greater reduction than the natural nitrate source [7]. This result is thought to be due to the nitrite being added directly to the sausage batters. Conversely, the number of *L. monocytogenes* decreased in all groups as the ripening time increased, but as can be seen in Figure 5, there was a slight decrease in groups C and D and group A on day 3. In contrast, on the same day, there was no change in the number in the group cured with natural nitrate (group B). However, on day 5, the number of *L. monocytogenes* decreased in this group. Similarly, in a study on salami by Hunt et al. [10], it was reported that synthetic nitrite and SCP showed similar results on days 3 and 7 of ripening. In the present study, a decrease in the number of *L. monocytogenes* was observed in all groups as ripening progressed. At the end of ripening, no significant difference was observed in the number of *L. monocytogenes* between the groups (Figure 5). These results indicate that SCP and pre-converted SCP have similar effects on the behavior of *L. monocytogenes* to those of synthetic curing agents. However, applying appropriate process conditions, regardless of the source of the curing agent in the sucuk production process, is of great importance. Since nitrite is the true curing agent, nitrate must first be converted to nitrite [4]. Indeed, Sebranek [60] and Gassara et al. [61] also reported that nitrite plays a role in the inhibition of pathogenic microorganisms, including *L. monocytogenes*.

As can be seen from Figure 5, it was not possible to eliminate *L. monocytogenes* from the sucuk in any group. Indeed, a study conducted on sucuk reported that as the ripening period progressed, the number of *L. monocytogenes* decreased in the presence of starter culture, but it was not possible to eliminate this foodborne pathogenic microorganism from the product [31]. Studies on other types of fermented sausages have also reported that *L. monocytogenes* survives in dry fermented sausages despite hurdle effects such as low pH, a_w_, competitive flora, and nitrite [26,62,63,64]. A study conducted by Erol and Hildebrant [35] also reported that *L. monocytogenes* showed slower growth at a ripening temperature of 20 °C compared to 25 °C, but this growth was limited by the use of starter cultures. Erol et al. [65] reported that the presence of bacteriocin-producing strains (*P. acidilactici* PAC 1.0 and *L. sakei* Lb 706) in sucuk ripened for 14 days at 20 °C and 25 °C led to a significant reduction in the number of *L. monocytogenes*. Kaya and Gökalp [32] also found that starter cultures, especially bacteriocin-producing lactic acid bacteria strains, caused a significant reduction in bacterial count during the ripening of sucuk. Coşansu et al. [66] also reported a significant reduction in *L. monocytogenes* numbers in the presence of a bacteriocin-producing strain. In the same study, the reduction rate in the control group was determined to be 1.37. A similar reduction rate was determined in the present study (Table 4). Kamiloğlu et al. [33] reported that a slight increase in the number of *L. monocytogenes* was observed on the first day of fermentation in sucuk produced without starter culture, but the number decreased as time progressed. In this control group, a reduction of 0.54 log cfu/g was observed at the end of ripening. In addition, a decrease of 2.74 log cfu/g was detected at the end of ripening in the presence of *L. plantarum* S50, which showed antagonistic activity against *L. monocytogenes*. Hampikyan and Uğur [67] also reported that the inhibition of *L. monocytogenes* increased with increasing nisin concentration in sucuk.

## 4. Conclusions

This study indicated that SCP used in slow ripening and pre-converted SCP used in fast ripening showed similar results to synthetic nitrite and nitrate with regard to the general characteristics of the product. The number of *L. monocytogenes* decreased with increasing ripening time in both slow and fast ripening. At the end of ripening, there were no significant differences in the number of *L. monocytogenes* in any group. Therefore, it has been concluded that SCP and pre-converted SCP can be used as alternative curing agents in sucuk production. Furthermore, these products can be an alternative to meet consumers’ demand for natural products. In industrial applications, however, it is necessary to check whether the nitrite content in the pre-converted products is sufficient. It should also be noted that the nitrate content in vegetables varies depending on climatic and geographical conditions. In addition, further studies on the application of hurdle technology to inhibit foodborne pathogens are considered important.

## Figures and Tables

**Table 1:** Microbiological enumerations.

Microorganism	Method	Culture Medium	Incubation Conditions	Evaluation	Reference
Lactic acid bacteria	Spread plate	MRS-Agar (Merck, Darmstadt, Germany)	30 °C, 48 h Anaerobic (Anaerocult A, Merck)	Gram (+), catalase (−)	Akköse et al. [29]
*Micrococcus*/*Staphylococcus*	Spread plate	MSA-Agar (Merck)	30 °C, 48 h Aerobic	Gram (+), catalase (+) cocci	Akköse et al. [29]
*Enterobacteriaceae*	Spread plate	VRBD-Agar (Merck)	30 °C, 48 h Anaerobic (Anaerocult A, Merck)	Rose red colonies larger than 1 mm	Yılmaz Oral and Sallan [40]
*Listeria monocytogenes*	Spread plate	PALCAM Listeria Selective-Agar (Merck)	37 °C, 48 h Aerobic	Green-black colored colonies Gram (+), catalase (+) Motile (22 °C)	ISO [41]

**Table 2:** Overall effect of curing agent (treatment) and ripening time on pH, a_w_ and residual nitrite content of sucuk.

Factors	N	pH	a_w_	Residual Nitrite (mg/kg) ^1^
Type of Curing Agent (TCA)				
A	21	5.11 ± 0.51 b	0.935 ± 0.029 a	8.82 ± 1.27 a
B	21	5.20 ± 0.49 a	0.930 ± 0.031 b	8.66 ± 1.28 a
C	21	4.94 ± 0.36 c	0.926 ± 0.032 c	9.16 ± 2.75 a
D	21	5.09 ± 0.40 b	0.914 ± 0.035 d	9.43 ± 1.16 a
Significance		**	**	NS
Ripening Time (RT)				
0	12	5.86 ± 0.06 a	0.964 ± 0.004 a	-
1	12	5.56 ± 0.43 b	0.962 ± 0.006 b	-
3	12	4.99 ± 0.19 c	0.947 ± 0.008 c	-
5	12	4.81 ± 0.09 d	0.927 ± 0.009 d	-
7	12	4.80 ± 0.06 d	0.913 ± 0.012 e	-
9	12	4.79 ± 0.06 d	0.896 ± 0.010 f	-
11	12	4.80 ± 0.07 d	0.877 ± 0.011 g	-
Significance		**	**	
TCA × RT		**	**	

A: 150 mg/kg KNO_3_; B: 150 mg/kg KNO_3_ equivalent SCP; C: 150 mg/kg NaNO_2_; D: 150 mg/kg NaNO_2_ equivalent pre-converted SCP. a–g: Any two means in the same column having the same letters in the same section are not significantly different at *p* > 0.05; ** *p* < 0.01; NS: not significant. ^1^ Residual nitrite content was only examined in the final product.

**Table 3:** Overall effect of the curing agent (treatment) and ripening time on the counts of lactic acid bacteria and *Micrococcus*/*Staphylococcus* of sucuk (log cfu/g).

Factors	N	Lactic Acid Bacteria	*Microccoccus*/*Staphylococcus*
Type of Curing Agent (TCA)			
A	21	8.07 ± 0.56 c	6.67 ± 0.39 a
B	21	7.82 ± 0.39 d	6.61 ± 0.41 a
C	21	8.49 ± 0.48 a	6.29 ± 0.30 b
D	21	8.19 ± 0.46 b	6.27 ± 0.21 b
Significance		**	**
Ripening Time (RT)			
0	12	7.35 ± 0.20 d	6.31 ± 0.20 c
1	12	8.05 ± 0.74 c	6.48 ± 0.23 b
3	12	8.54 ± 0.28 a	6.69 ± 0.41 a
5	12	8.54 ± 0.30 a	6.73 ± 0.49 a
7	12	8.38 ± 0.28 b	6.53 ± 0.35 b
9	12	8.13 ± 0.28 c	6.33 ± 0.29 c
11	12	8.01 ± 0.26 c	6.15 ± 0.32 d
Significance		**	**
TCA × RT		**	**

A: 150 mg/kg KNO_3_; B: 150 mg/kg KNO_3_ equivalent SCP; C: 150 mg/kg NaNO_2_; D: 150 mg/kg NaNO_2_ equivalent pre-converted SCP. a–d: Any two means in the same column having the same letters in the same section are not significantly different at *p* > 0.05; ** *p* < 0.01; NS: not significant.

**Table 4:** Overall effect of the curing agent (treatment) and ripening time on the counts of *Listeria monocytogenes* of sucuk.

Factors	N	*Listeria monocytogenes* (log cfu/g)
Type of Curing Agent (TCA)		
A	21	3.25 ± 0.50 a
B	21	3.32 ± 0.36 a
C	21	3.37 ± 0.50 a
D	21	3.31 ± 0.50 a
Significance		NS
Ripening Time (RT)		
0	12	4.00 ± 0.13 a
1	12	3.77 ± 0.28 b
3	12	3.33 ± 0.25 c
5	12	3.32 ± 0.10 c
7	12	3.08 ± 0.22 d
9	12	2.89 ± 0.18 e
11	12	2.77 ± 0.18 e
Significance		**
TCA × RT		**

A: 150 mg/kg KNO_3_; B: 150 mg/kg KNO_3_ equivalent SCP; C: 150 mg/kg NaNO_2_; D: 150 mg/kg NaNO_2_ equivalent pre-converted SCP. a–e: Any two means in the same column having the same letters in the same section are not significantly different at *p* > 0.05; ** *p* < 0.01; NS: not significant.

## Data Availability

The original contributions presented in this study are included in the article/Supplementary Material, further inquiries can be directed to the corresponding author.

## Supplementary Materials

The following supporting information can be downloaded at: https://www.mdpi.com/article/10.3390/foods15152728/s1, Table S1: Experimental plan.

## References

[1] Tabanelli G., Barbieri F., Soglia F., Magnani R., Gardini G., Petracci M., Montanari C. (2022). Safety and technological issues of dry fermented sausages produced without nitrate and nitrite. Food Res. Int..

[2] Kim M., Bae S.M., Yoo Y., Park J., Jeong J.Y. (2025). Clean-label strategies for the replacement of nitrite, ascorbate, and phosphate in meat products: A review. Foods.

[3] Honikel K.O. (2008). The use and control of nitrate and nitrite for the processing of meat products. Meat Sci..

[4] Alahakoon A.U., Jayasena D.D., Ramachandra S., Jo C. (2015). Alternatives to nitrite in processed meat: Up to date. Trends Food Sci. Technol..

[5] Ferysiuk K., Wójciak K.M. (2020). Reduction of nitrite in meat products through the application of various plant-based ingredients. Antioxidants.

[6] Kaya M., Kaban G., Aran N. (2019). Fermente et ürünleri. Gıda Biyoteknolojisi.

[7] Dalzini E., Merigo D., Caproli A., Monastero P., Cosciani-Cunico E., Losio M.N., Daminelli P. (2022). Inactivation of *Listeria monocytogenes* and *Salmonella* spp. in milano-type salami made with alternative formulations to the use of synthetic nitrates/nitrites. Microorganisms.

[8] Sebranek J.G., Bacus J.N. (2007). Cured meat products without direct addition of nitrate or nitrite: What are the issues?. Meat Sci..

[9] Fraqueza M.J., Laranjo M., Elias M., Patarata L. (2021). Microbiological hazards associated with salt and nitrite reduction in cured meat products: Control strategies based on antimicrobial effect of natural ingredients and protective microbiota. Curr. Opin. Food Sci..

[10] Hunt H.B., Mills E., Cutter C.N., Campbell J., Cutter C. (2025). Fate of pathogens during fermentation, drying, and storage of salami cured with various sources of nitrite. Meat Muscle Biol..

[11] Flores M., Toldrá F. (2021). Chemistry, safety, and regulatory considerations in the use of nitrite and nitrate from natural origin in meat products-Invited review. Meat Sci..

[12] Jo K., Lee S., Yong H.I., Choi Y.S., Jung S. (2020). Nitrite sources for cured meat products. LWT-Food Sci. Tech..

[13] Magrinya N., Bou R., Tres A., Rius N., Codony R., Guardiola F. (2009). Effect of tocopherol extract, *Staphylococcus carnosus* culture, and celery concentrate addition on quality parameters of organic and conventional dry-cured sausages. J. Agricul. Food Chem..

[14] Bertol T.M., Fiorentini A.M., Santos M.J.H.D., Sawitzki M.C., Kawski V.L., Agnes I.B.L., Costa C.D., Coldebella A., Lopes L.D.S. (2012). Rosemary extract and celery-based products used as natural quality enhancers for colonial type salami with different ripening times. Food Sci. Technol..

[15] Tsoukalas D.S., Katsanidis E., Marantidou S., Bloukas J.G. (2011). Effect of freeze-dried leek powder (FDLP) and nitrite level on processing and quality characteristics of fermented sausages. Meat Sci..

[16] Eisinaite V., Vinauskiene R., Viskelis P., Leskauskaite D. (2016). Effects of freeze-dried vegetable products on the technological process and the quality of dry fermented sausages. J. Food Sci..

[17] Sucu C., Turp G.Y. (2018). The investigation of the use of beetroot powder in Turkish fermented beef sausage (sucuk) as nitrite alternative. Meat Sci..

[18] Martínez L., Bastida P., Castillo J., Ros G., Nieto G. (2019). Green alternatives to synthetic antioxidants, antimicrobials, nitrates, and nitrites in clean label Spanish chorizo. Antioxidants.

[19] Ozaki M.M., Munekata P.E., de Souza Lopes A., do Nascimento M.D.S., Pateiro M., Lorenzo J.M., Pollonio M.A.R. (2020). Using chitosan and radish powder to improve stability of fermented cooked sausages. Meat Sci..

[20] Pennisi L., Verrocchi E., Paludi D., Vergara A. (2020). Effects of vegetable powders as nitrite alternative in Italian dry fermented sausage. Ital. J. Food Saf..

[21] Ozaki M.M., Munekata P.E., Jacinto-Valderrama R.A., Efraim P., Pateiro M., Lorenzo J.M., Pollonio M.A.R. (2021). Beetroot and radish powders as natural nitrite source for fermented dry sausages. Meat Sci..

[22] Babaoğlu A.S., Karakaya M. (2022). Investigation of the quality characteristics of naturally cured sucuks with dill, spinach and Swiss chard powders during refrigerated storage. Selçuk J. Agric. Food Sci..

[23] Yılmaz Oral Z.F. (2023). The effect of using Swiss chard powder as a curing agent in the production of heat-treated sucuk on nitrosamine formation and quality parameters. GIDA.

[24] Engin N. (2022). Isıl işlem görmüş sucukta kereviz tozu kullanımının *Listeria monocytogenes*’ in davranışına etkisi. Y.Lisans Tezi.

[25] Fernandez M., Hospital X.F., Caballero N., Jiménez B., Sánchez-Martín V., Morales P., Hierro E. (2023). Potential of selected bacteriocinogenic lactic acid bacteria to control *Listeria monocytogenes* in nitrite-reduced fermented sausages. Food Control.

[26] Christieans S., Picgirard L., Parafita E., Lebert A., Gregori T. (2018). Impact of reducing nitrate/nitrite levels on the behavior of *Salmonella* Typhimurium and *Listeria monocytogenes* in French dry fermented sausages. Meat Sci..

[27] Hospital X.F., Fernandez M., Herranz C., Martin-Cabrejas I., Caballero N., Jimenez B., Hierro E. (2025). Control of *Listeria monocytogenes* in Nitrite-Reduced Mediterranean Dry-Fermented Sausages Using Two Bacteriocinogenic *Pediococcus acidilactici* Strains. Food Bioprocess Technol..

[28] Soyer A., Ertaş A.H., Üzümcüoğlu Ü. (2005). Effect of processing conditions on the quality of naturally fermented Turkish sausages (sucuks). Meat Sci..

[29] Akköse A., Şişik Oğraş Ş., Kaya M., Kaban G. (2023). Microbiological, physicochemical and sensorial changes during the ripening of sucuk, a traditional Turkish dry-fermented sausage: Effects of autochthonous strains, sheep tail fat and ripening rate. Fermentation.

[30] Republic of Türkiye Ministry of Agriculture and Forestry (2023). Turkish Food Codex Regulation on Food Additives (2023).

[31] Kaya M., Gökalp H.Y. (2004). Sucuk üretiminde starter kültür kullanımının ve farklı nitrit dozlarının *Listeria monocytogenes’* in gelişimi üzerine etkisi. Turk. J. Vet. Anim. Sci..

[32] Kaya M., Gökalp H.Y. (2004). Farklı laktik starter kültürler kullanılarak üretilen sucuklarda *Listeria monocytogenes*’ in davranışı. Turk. J. Vet. Anim. Sci..

[33] Kamiloğlu A., Kaban G., Kaya M. (2019). Effects of autochthonous *Lactobacillus plantarum* strains on *Listeria monocytogenes* in sucuk during ripening. J. Food Saf..

[34] Yılmaz Topcam M.M., Arslan B., Soyer A. (2024). Sucuk, Turkish-style fermented sausage: Evaluation of the effect of bioprotective starter cultures on its microbiological, physicochemical, and chemical properties. Appl. Microbiol..

[35] Erol İ., Hildebrandt G. (1992). Influence of starter cultures on the growth of pathogenic organisms in Turkısh dry sausage. Fleischwirtschaft.

[36] Yılmaz Oral Z.F. (2023). Effect of celery powder as an alternative nitrite source on some quality properties and nitrosamine formation in sucuk. Kafkas Univ. Vet. Fak. Derg..

[37] Kaban G., Kaya M. (2008). Identification of lactic acid bacteria and gram-positive catalase-positive cocci ısolated from naturally fermented sausage (Sucuk). J. Food Sci..

[38] Kaya M., Sayın B., Topçu K.Ç., Karadayı M., Kamiloğlu A., Güllüce M., Kaban G. (2025). Genotypic and technological characterization of lactic acid bacteria and coagulase-negative staphylococci isolated from Sucuk: A preliminary screening of potential starter cultures. Foods.

[39] NMKL (Nordic Committee of Food Analysis) (2000). Nitrite and Nitrate in Foodstuffs by Ion Chromatography.

[40] Yılmaz Oral Z.F., Sallan S. (2023). Evaluation of quality characteristics of commercial fermented sausages (sucuk and heat-treated sucuk). Turk. J. Agric.-Food Sci. Technol..

[41] (2017). Microbiology of the Food Chain—Horizontal Method for the Detection and Enumeration of Listeria monocytogenes and of Listeria spp.—Part 1: Detection Method.

[42] Lücke F.K., Wood B.J.B. (1998). Fermented sausages. Microbiology of Fermented Foods.

[43] Toldra F., Sanz Y., Flores M., Hui Y.H., Nip W.K., Rogers R.W., Young O.A. (2001). Meat fermentation technology. Meat Science and Applications.

[44] Caplice E., Fitzgerald G.G. (1999). Food fermentations: Role of microorganisms in food production and preservation. Int. J. Food Microbiol..

[45] Papadima S.N., Bloukas J.G. (1999). Effect of fat level and storage conditions on quality characteristics of traditional Greek sausages. Meat Sci..

[46] European Commission (EC) Commission Regulation (EC) No. 2073/2005 of 15 November 2005 on microbiological criteriafor foodstuffs. Official Journal of the European Union, L338, 2005. https://eur-lex.europa.eu/eli/reg/2005/2073/oj/eng.

[47] Djeri N. (2010). Evaluation of Veg Stable^TM^ 504 Celery Juice Powder for Use in Processed Meat and Poultry as a Nitrite Replacer. Ph.D. Thesis.

[48] Yurchenko S., Mölder U. (2007). The occurrence of volatile N-nitrosamines in Estonian meat products. Food Chem..

[49] Molognoni L., Motta G.E., Daguer H., Lindner J.D.D. (2020). Microbial biotransformation of N-nitro-, C-nitro-, and C-nitrous-type mutagens by *Lactobacillus delbrueckii* subsp. bulgaricus in meat products. Food Chem. Toxicol..

[50] García-Díez J., Saraiva C. (2021). Use of Starter Cultures in Foods from Animal Origin to Improve Their Safety. Int. J. Environ. Res. Public Health.

[51] Hospital X.F., Carballo J., Fernández M., Arnau J., Gratacós M., Hierro E. (2015). Technological implications of reducing nitrate and nitrite levels in dry-fermented sausages: Typical microbiota, residual nitrate and nitrite and volatile profile. Food Control.

[52] Chen X., Mi R., Qi B., Xiong S., Li J., Qu C., Wang S. (2021). Effect of proteolytic starter culture isolated from Chinese Dong fermented pork (Nanx Wudl) on microbiological, biochemical and organoleptic attributes in dry fermented sausages. Food Sci. Hum. Wellness.

[53] Stegmayer M.A., Sirini N.E., Ruiz M.J., Soto L.P., Zbrun M.V., Lorenzo J.M., Frizzo L.S. (2023). Effects of lactic acid bacteria and coagulase-negative staphylococci on dry-fermented sausage quality and safety: Systematic review and meta-analysis. Meat sci..

[54] Castano A., Fontán M.G., Fresno J.M., Tornadijo M.E., Carballo J. (2002). Survival of Enterobacteriaceae during processing of Chorizo de cebolla, a Spanish fermented sausage. Food Control.

[55] Paramithiotis S., Drosinos E.H., Zdolec N. (2017). Microbial Spoilage of Fermented Meat Products. Fermented Meat Products-Health Aspects.

[56] Stegmayer M.Á., Sirini N.E., Soto L.P., Zimmermann J.A., Zbrun M.V., Lencina F.A., Frizzo L.S. (2025). Effect of NaCl reduction and/or replacement on the quality and safety of dry-fermented sausages: Systematic review and meta-analysis. Meat Sci..

[57] Golden M.C., McDonnell L.M., Sheehan V., Sindelar J.J., Glass K.A. (2010). Inhibition of *Listeria monocytogenes* in deli-style Turkey breast formulated with cultured celery powder and/or cultured sugar–vinegar blend during storage at 4 °C. J. Food Protec..

[58] Modzelewska-Kapituła M., Lemański A., Zduńczyk W., Zadernowska A. (2022). Investigation of the Possibility of *Listeria monocytogenes* Growth in Alternatively Cured Cooked Sausages—A Case Study. Appl. Sci..

[59] Riel G., Boulaaba A., Popp J., Klein G. (2017). Effects of parsley extract powder as an alternative for the direct addition of sodium nitrite in the production of mortadellatype sausages–Impact on microbiological, physicochemical and sensory aspects. Meat Sci..

[60] Sebranek J.G., Tarte R. (2009). Basic curing ingredients. Gredients in Meat Products: Properties, Functionality and Applications.

[61] Gassara F., Kouassi A.P., Brar S.K., Belkacemi K. (2016). Green alternatives to nitrates and nitrites in meat-based products—A review. Crit. Rev. Food Sci. Nutr..

[62] Nissen H., Holck A. (1998). Survival of *Escherichia coli* O157: H7, *Listeria monocytogenes* and *Salmonella* kentuckyin Norwegian fermented, dry sausage. Food Microbiol..

[63] Barbuti S., Parolari G. (2002). Validation of manufacturing process to control pathogenic bacteria in typical dry fermented products. Meat Sci..

[64] Meloni D. (2015). Presence of *Listeria monocytogenes* in Mediterranean-style dry fermented sausages. Foods.

[65] Erol İ., Çelik T.H., Şireli U.T., Özdemir H. (1999). Bakteriyosin oluşturan starter kültürlerin fermente Türk sucuklarında *Listeria monocytogenes* üzerine etkisi. Turk. J. Vet. Anim. Sci..

[66] Coşansu S., Geornaras I., Ayhan K., Sofos J.N. (2010). Control of *Listeria monocytogenes* by bacteriocin-producing *Pediococcus acidilactici* 13 and its antimicrobial substance in a dry fermented sausage sucuk and in turkey breast. J. Food Nutr. Res..

[67] Hampikyan H., Ugur M. (2007). The effect of nisin on *L. monocytogenes* in Turkish fermented sausages (sucuks). Meat Sci..

