# Supplementary material for: Effect of Using Swiss Chard Powder as Alternative Curing Agent in Sucuk Production on the Growth of Listeria monocytogenes
Source: Foods. 2026 Aug 3;15(15):2728. doi: 10.3390/foods15152728 (PMC13465773; doi:10.3390/foods15152728)
Supplement: Supplementary file 1 [file foods-15-02728-s001.zip › foods-4444873-supplementary.pdf]

Table S1. Experimental plan

Type of Curing Agent	Block (biological replication)	Ripening Time (day)						
		0	1	3	5	7	9	11
A	1							
	2							
	3							
B	1							
	2							
	3							
C	1							
	2							
	3							
D	1							
	2							
	3							

A: 150 mg/kg KNO_3; B: 150 mg/kg KNO_3 equivalent SCP; C: 150 mg/kg $NaNO_2$; D: 150 mg/kg $NaNO_2$ equivalent pre-converted SCP
